# Supplementary material for: bHLH010/089 Transcription Factors Control Pollen Wall Development via Specific Transcriptional and Metabolic Networks in Arabidopsis thaliana
Source: Int J Mol Sci. 2022 Oct 2;23(19):11683. doi: 10.3390/ijms231911683 (PMC9570398; doi:10.3390/ijms231911683)
Supplement: Supplementary file 1 [file ijms-23-11683-s001.zip › ijms-1898779-supplementary.pdf]

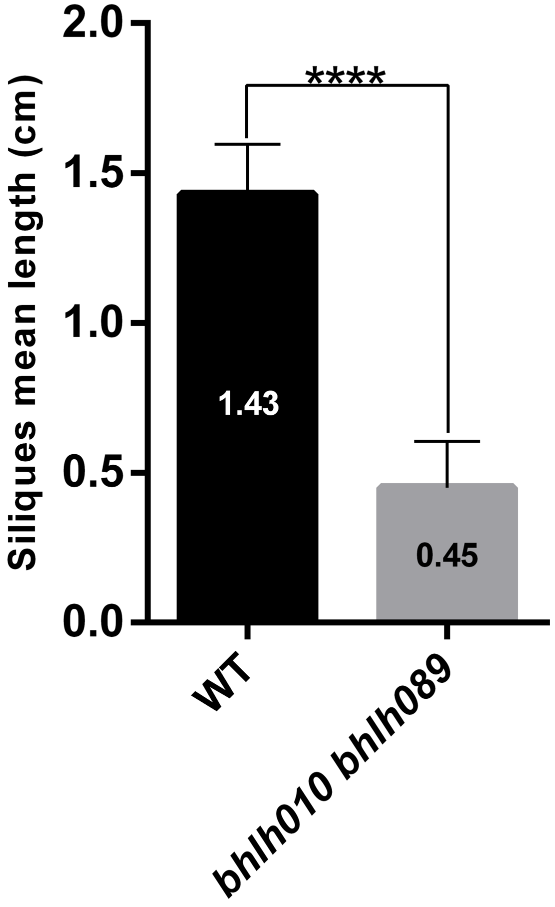

Supplemental Figure S1. The silique length of *bhlh010 bhlh089* mutant plants was obviously reduced.
Student's *t*-test was used for difference comparison. ****, P<0.0001. Data were means ± SD (n =3).

Lai_Fig S2

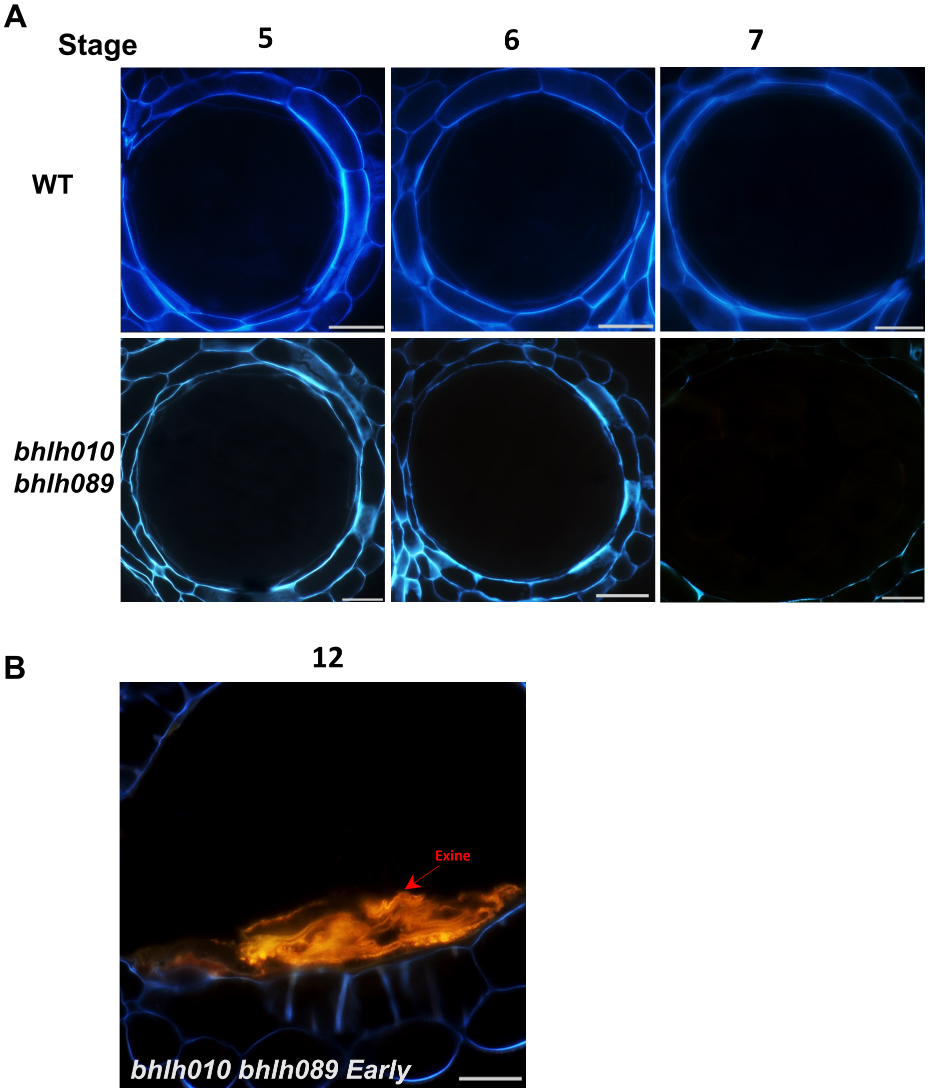

Supplemental Figure S2. Transverse sections of the pollen wall development were performed in WT and the *bhlh010 bhlh089* double mutant .

(A) The development of pollen wall has not yet begun at stage 5-7. There was no significant difference between WT and *bhlh010 bhlh089* double mutant. Bars =10 um.

(B) The pollen of the first 12 flowers were completely aborted. It only forms the pollen exine, not intine. The red arrow indicates the exine. Bars =10 um.

Lai_Fig S3

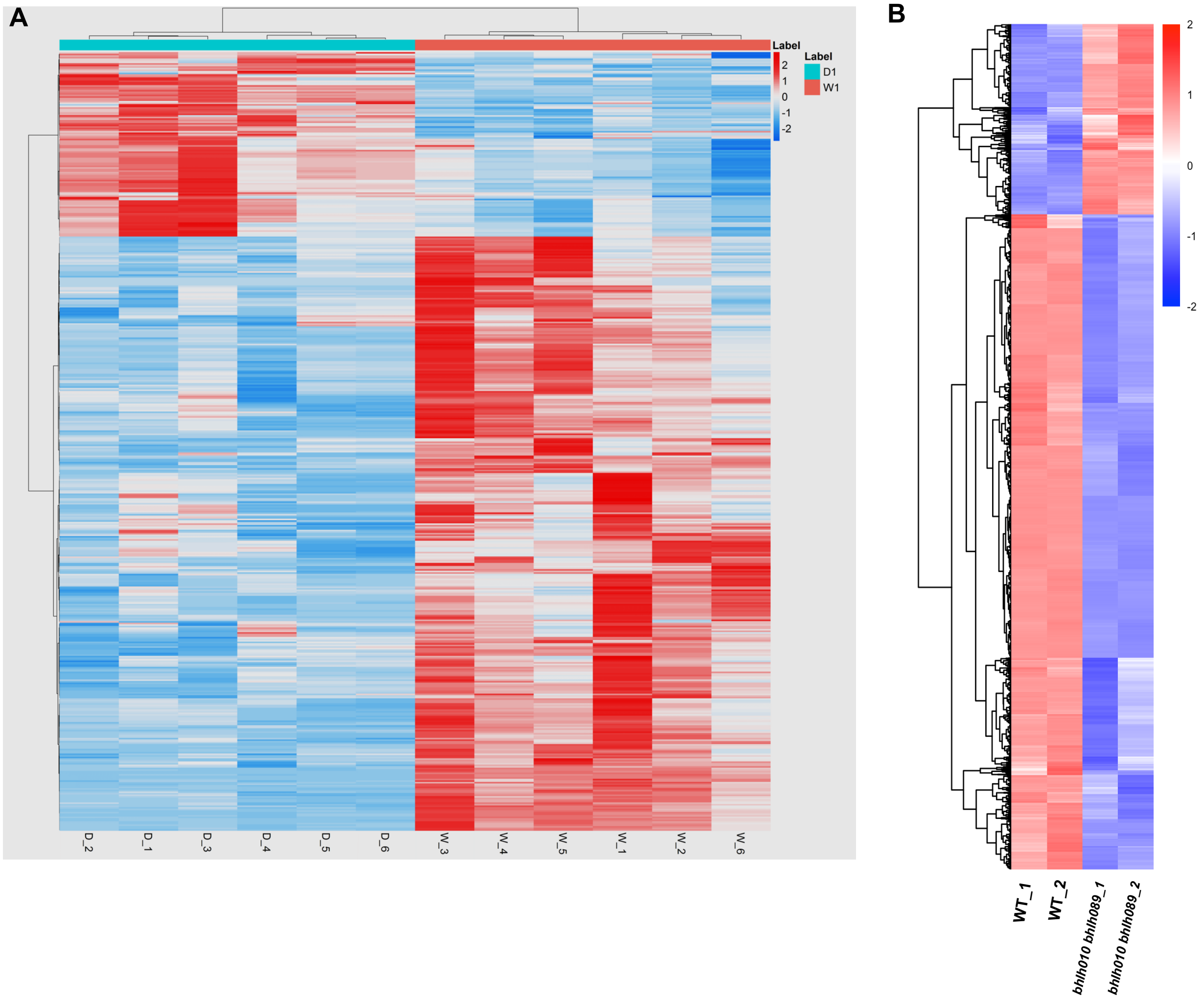

Supplemental Figure S3. Analysis of total differential genes and total differential metabolites of WT and *bhlh010 089* double mutants.

(A) The Heat maps represented total differential metabolites (DEMs) involved in WT (W1) and *bhlh010 bhlh089* (D1) double mutant inflorescence . Differential metabolites were screened out by VIP (Variable Importance in the Projection) value of OPLS-DA model (VIP >= 1) and independent sample *t*-test ($p < 0.05$). Adduct manner: $[M+H]^+$ and $[M+Na]^+$ was selected in positive mode, $[M-H]^-$ in negative mode. Mass error value: 30 PPM. red and blue color represents upregulated and downregulated DEMs.

(B) The Heat maps represented total differential genes (DEGs) involved in WT and *bhlh010 bhlh089* double mutant anthers. Relative expression was calculated by $-\log_2$ (Fold change) and $P < 0.01$, red and blue color represents upregulated and downregulated DEGs.

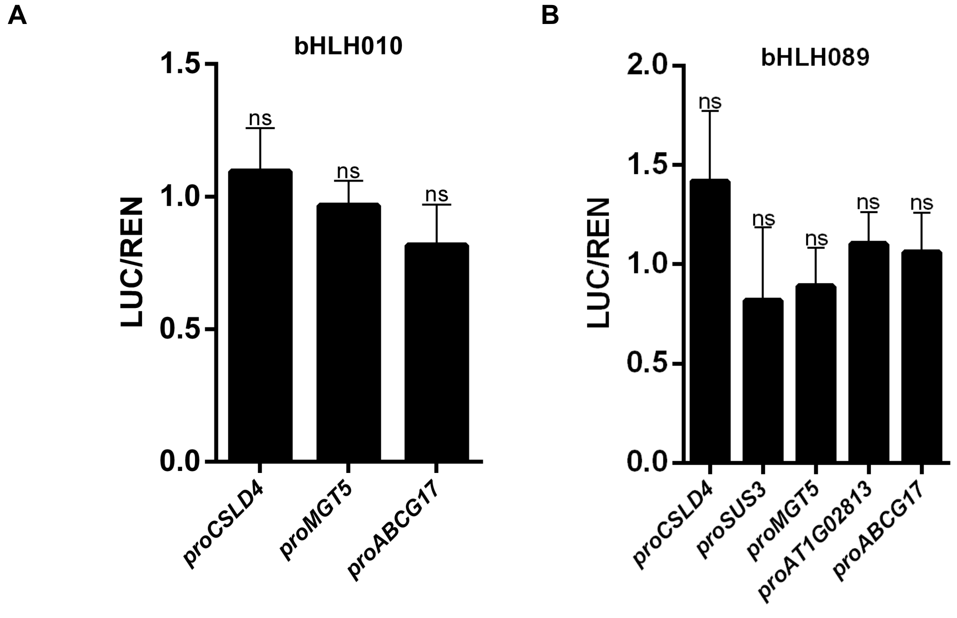

Supplemental Figure S4. The transcriptional activation capacity of bHLH010 and bHLH089 was detected.

(A, B) Dual-Luciferase assay was used to detect transcriptional activation. **(A)** The transcription factor bHLH010 does not directly transcribes activated genes. **(B)** The transcription factor bHLH089 does not directly transcribes activated genes. Significant differences compared with the WT were determined using Student's *t*-test: ns indicates no significant difference. Data are shown as means±SD (n =3).

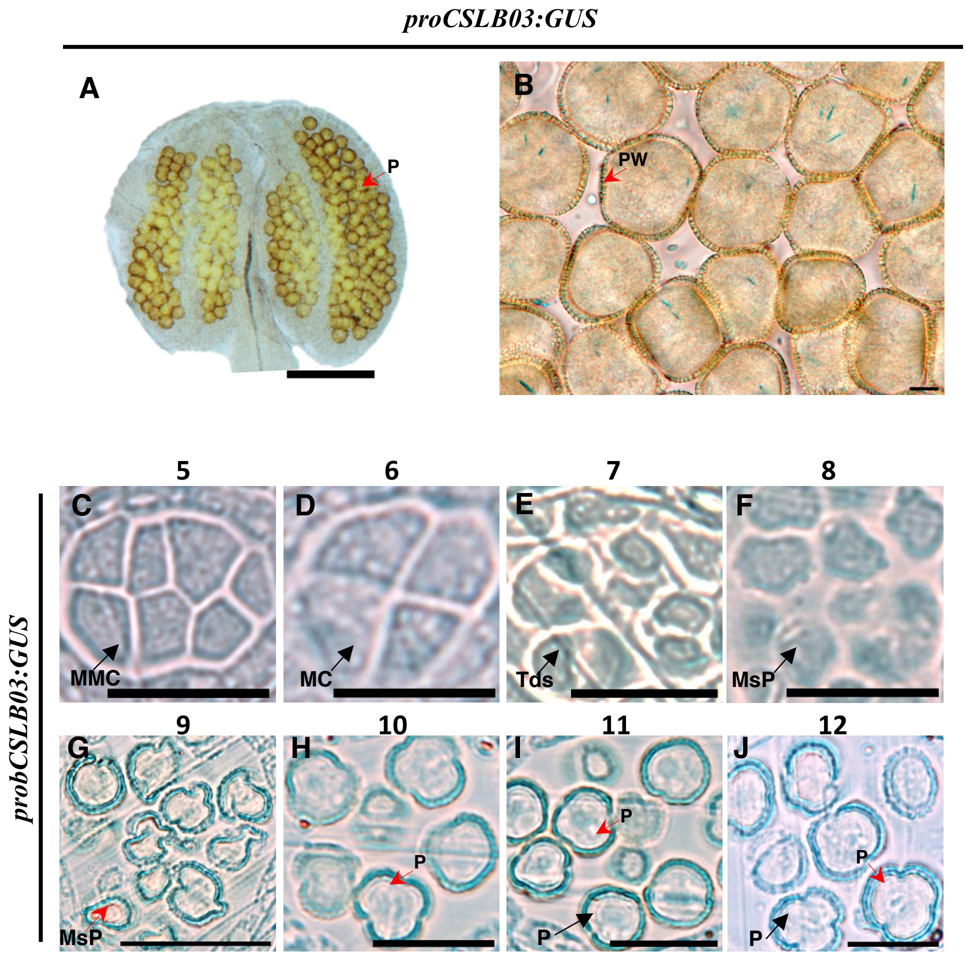

Supplemental Figure S5. Tissue expression pattern of CSLB03.

(A) Tissue expression pattern of CSLB03 in *proCSLB03:GUS*, bar=50 μm. CSLB03 is specifically expressed in the pollen of anthers. P, pollen. bar=50 μm.

(B) CSLB03 is specifically expressed in the pollen. PW, pollen wall. bar=10 μm.

(C-J) Transverse sections of the pollen development of CSLB03 in *proCSLB03:GUS* at stages 5–12. The CSLB03 was specially expressed in pollen. The CSLB03 expression level was highest in stage 9-12. MMC, Microspore mother cell ;MC, Microspore cell ;Tds, tetrad cells ; Msp, microspore; P, pollen; PW, pollen wall. Bars =25 um.

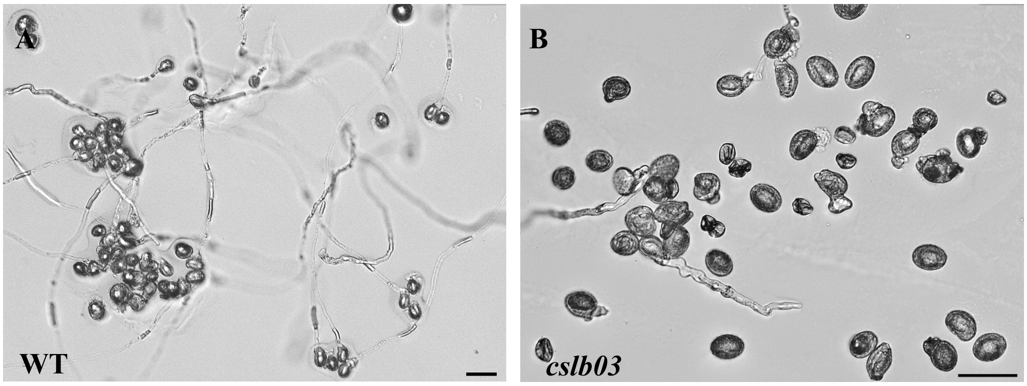

Supplemental Figure S6. The pollen germination of *cslb03* mutant was severely abnormal.

(A, B) Pollen germination experiment. Pollen germination of WT **(A)** was normal and that of *cslb03* mutant **(B)** could not completely germinate or germination was slow. Bars =50 μm.

Lai_Fig S7

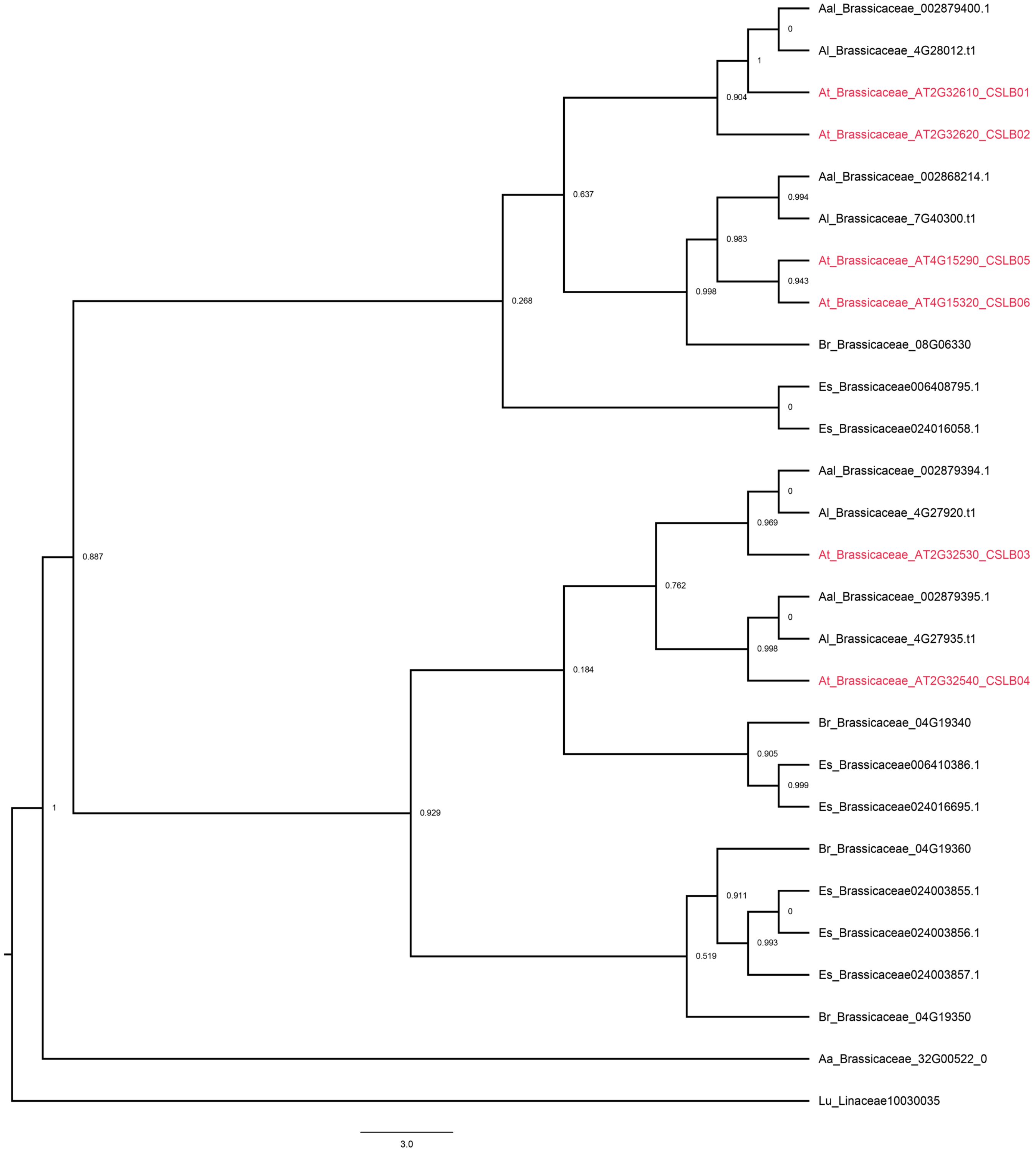

Supplemental Figure S7. Phylogenetic analysis of *CSLB1/2/3/4/5/6* subfamily.
The bootstrap values are indicated near the nodes. Genes from Arabidopsis thaliana are highlighted in red.
Aa, Aethionema arabicum ; Aal, Arabis alpine; At, Arabidopsis thaliana; Br, Brassica rapa; Es, Eutrema salsugineum; Lu, Linum usitatissimum.
